# Single-Cell Analysis Identify Transcription Factor BACH1 as a Master Regulator Gene in Vascular Cells During Aging

**DOI:** 10.3389/fcell.2021.786496

**Published:** 2021-12-24

**Authors:** Fei Ge, Qi Pan, Yue Qin, Mengping Jia, Chengchao Ruan, Xiangxiang Wei, Qing Jing, Xiuling Zhi, Xinhong Wang, Lindi Jiang, Elena Osto, Jieyu Guo, Dan Meng

**Affiliations:** ^1^ Department of Physiology and Pathophysiology, School of Basic Medical Sciences, Department of Rheumatology, Zhongshan Hospital, Fudan University, Shanghai, China; ^2^ CAS Key Laboratory of Tissue Microenvironment and Tumor, Shanghai Institute of Nutrition and Health, Shanghai Institutes for Biological Sciences, University of Chinese Academy of Sciences, Chinese Academy of Sciences, Shanghai, China; ^3^ Institute of Clinical Chemistry and Department of Cardiology, University Heart Center, University and University Hospital Zurich, Zurich, Switzerland

**Keywords:** vascular aging, endothelial cells, BACH1, scRNA-seq, cellular heterogeneity

## Abstract

Vascular aging is a potent driver of cardiovascular and cerebrovascular diseases. Vascular aging features cellular and functional changes, while its molecular mechanisms and the cell heterogeneity are poorly understood. This study aims to 1) explore the cellular and molecular properties of aged cardiac vasculature in monkey and mouse and 2) demonstrate the role of transcription factor BACH1 in the regulation of endothelial cell (EC) senescence and its mechanisms. Here we analyzed published single-cell RNA sequencing (scRNA-seq) data from monkey coronary arteries and aortic arches and mouse hearts. We revealed that the gene expression of YAP1, insulin receptor, and VEGF receptor 2 was downregulated in both aged ECs of coronary arteries’ of monkey and aged cardiac capillary ECs of mouse, and proliferation-related cardiac capillary ECs were significantly decreased in aged mouse. Increased interaction of ECs and immunocytes was observed in aged vasculature of both monkey and mouse. Gene regulatory network analysis identified BACH1 as a master regulator of aging-related genes in both coronary and aorta ECs of monkey and cardiac ECs of mouse. The expression of BACH1 was upregulated in aged cardiac ECs and aortas of mouse. BACH1 aggravated endothelial cell senescence under oxidative stress. Mechanistically, BACH1 occupied at regions of open chromatin and bound to *CDKN1A* (encoding for P21) gene enhancers, activating its transcription in senescent human umbilical vein endothelial cells (HUVECs). Thus, these findings demonstrate that BACH1 plays an important role in endothelial cell senescence and vascular aging.

## Introduction

Aging is a major and independent risk factor for the occurrence of cardiovascular and cerebrovascular disease (Camici et al., 2015). The process of aging results in alterations in vascular structure and function, including increased media and intima stiffness, decreased elasticity, endothelial dysfunction, impaired angiogenesis, defective vascular repair, as well as enhanced inflammatory responses (Erusalimsky, 2009; Chen et al., 2016). Vascular aging is a key contributor of the progressive deterioration of organ function (Grunewald et al., 2021). The ever-growing elderly population highlights the need to better understand cellular and functional changes in the aging vasculature (Paneni et al., 2017) and their underlying mechanisms. Vascular aging is associated with multiple disarrangements including oxidative stress, mitochondrial dysfunction, impaired resistance to molecular stressors, and chronic low-grade inflammation (Ungvari et al., 2018). Many of these observations arise from aging modeling in mice or rats, but appraisal of interspecies differences in a model closer to humans is required in a translational view (Ungvari et al., 2018). Monkeys are similar to humans regarding their genetic, anato-physiological features, and their susceptibility to aging-related diseases (Zhang et al., 2020). Therefore, defining the signatures of aging responses in the vasculature among different species, including monkeys, is essential to elucidate the regulatory mechanisms of vascular aging with clinical relevance.

Transcription factor BTB and CNC homology 1 (BACH1) is a member of the basic leucine zipper (bZIP) protein family (Oyake et al., 1996). BACH1 is widely present in various mammalian tissues and is known for participation in oxidative stress response, cell cycle and differentiation, mitochondrial metabolism, and cancer metastasis (Zhang X. et al., 2018; Lee et al., 2019; Wiel et al., 2019). Under physiologic conditions, BACH1 binds to the small Maf proteins and acts on antioxidative response element (ARE) in gene promoters, inhibiting the transcription of many oxidative stress-response genes including glutathione S-transferase (GST) and heme oxygenase-1 (HO-1) (Dhakshinamoorthy et al., 2005). Under oxidative stress, BACH1 separates from ARE and exports from the nucleus. Its competitive factor, erythroid-derived nuclear factor-related factor 2 (NF-E2 related factor 2, Nrf2) combines with ARE, resulting in increased expression of antioxidant genes. Our previous studies have shown that BACH1 increases reactive oxygen species (ROS) generation in endothelial cells (ECs), induces EC apoptosis (Wang et al., 2016), reduces EC proliferation and migration, and suppresses angiogenic response to peripheral ischemic injury in adult mice (Jiang et al., 2015). Global knockout of BACH1 in mice was shown to attenuate the development of atherosclerosis and reduce the proliferation of vascular smooth muscle cells (SMCs) (Omura et al., 2005). Recently, a large-scale genome-wide association study (GWAS) also identified BACH1 as a human coronary artery disease (CAD)-related candidate gene (van der Harst and Verweij, 2018). Increased BACH1 expression has been found in the cerebellum, liver, and lung tissues of elderly mice (Zhang et al., 2012), and contributes to aging-related loss of Nrf2 signaling in primary human bronchial epithelial cells (Zhang et al., 2019). However, the role and underlying mechanisms of BACH1 in the regulation of vascular aging remains unclear.

Single-cell RNA sequencing (scRNA-seq) analysis can decipher cellular heterogeneity at single-cell level (Paik et al., 2020) and facilitate a better understanding of the patho-physiological role of functional heterogeneity of vascular aging. In the present study, we analyzed the changes in cell proportions and molecular properties in young and aged vessels using published scRNA-seq data from two datasets: 1) monkey coronary arteries and aortic arches; 2) mouse hearts. We showed that EC interaction with immune cells appears crucial in aged vessels of both monkeys and mice, and proliferation-related cardiac capillary ECs were significantly decreased in aged mouse. Gene regulatory network analysis identified BACH1 as a master regulator of aging-related genes in both coronary and aorta ECs of monkey and cardiac ECs of mouse. BACH1 was upregulated in aged cardiac ECs and aortas of mouse, and aggravated endothelial cell senescence under oxidative stress. Mechanistically, BACH1 occupies regions of open chromatin and binds to cyclin-dependent kinase inhibitor 1A (*CDKN1A*, encoding for P21) gene enhancers, activating its transcription in senescent human umbilical vein endothelial cells (HUVECs). Thus, BACH1 is essential in endothelial cell senescence and vascular aging.

## Materials and Methods

### Animals

All animal studies were approved by the Ethics Committee of Experimental Research at Fudan University. C57BL6/J mice were housed under a 12-h light/dark cycle in a pathogen-free condition with food and water available *ad libitum*. Two-month-old mice were classed as young group and 18-month-old mice were classed as old group. Mice were sacrificed and their aortic vessels were isolated for immunohistochemistry.

### Analysis of Published scRNA-seq Data

A published scRNA-seq dataset of aortic arches and coronary arteries from eight young (4–6 years old) and eight old (18–21 years old) monkeys (*Macaca fascicularis*) was analyzed (GEO accession no. GSE117715) (Zhang et al., 2020). Another published scRNA-seq dataset of heart vessels from male C57/B6 young (3 months, 13 animals) and aged (24 months, 13 animals) mice was also analyzed (GEO accession no. GSE163822) (Emechebe et al., 2021).

### Cell Clustering of scRNA-seq Data

Seurat (Version 3.2) package (Stuart et al., 2019) was used to analyze cellular and molecular components of vasculatures. First, the integrated objects were filtered, scaled (scale factor 100,000 for monkey and 10,000 for mouse) and normalized. Next, 2,000 highly variable genes were identified as inputs for principal component analysis. Significant principal components were then used to cluster cells at the resolution of 0.4 for monkey and 0.1 for mouse.

### Identification of Old versus Young differentially expressed genes

The edgeR package (Robinson et al., 2010) was used to normalize the data and compute differentially expressed genes (DEGs) of old cells and young cells from monkey and mouse, respectively. The expression matrix was first normalized, and then DEGs were defined (*p*-value ≤0.05 and |logFC| ≥ 0.5 for monkey, *p*-value ≤0.05 and |logFC| ≥ 0.25 for mouse). Seurat was also used to identify DEGs. DEGs with *p*_val_adj ≤0.05 and avg_logFC ≥0.5 were considered representative markers of different clusters.

### Transcription Factor Activity Analysis

Transcription factor (TF) activities of monkey and mouse were calculated by DoRothEA (https://github.com/saezlab/dorothea) (Holland et al., 2020). Transcription factor activities of highest difference from old and young cells were visualized through heatmaps.

### Ligand–Receptor Cellular Communication Analysis

Ligand-receptor cellular communication analysis of monkey and mouse were performed using CellPhoneDB (Efremova et al., 2020). Mean number of ligand–receptor interaction of different cell types was visualized through heatmaps.

### Network of BACH1 and Target Genes

Regulatory network analysis using R package GENIE3 was performed to determine key transcriptional regulators in aged ECs (Aibar et al., 2017). Old vs. young DEG expression matrix was used as input for network analysis to construct the network of aging-associated transcriptional regulators and targets. Transcriptional regulator–target connections with a high weight (>0.5) were used for the network analysis and visualized.

### Pseudotime Analysis for Signaling Pathway-Related Genes

CA_EC clusters were used to construct single-cell trajectories of cells for CA_EC-related clusters from a young state to an old state and perform pseudotime analysis using Monocle2 package (Qiu et al., 2017). First, DEGs identified by the Seurat package were set to ordering filter. Then,the “reduceDimension” based on DDRTree algorithm and “plot_pseudotime_heatmap” methods were used for pseudotime analysis. Furthermore, genes in aging-related signaling pathways (Hippo signaling pathway, insulin signaling pathway, and VEGF signaling pathway) were visualized through heatmap.

### ATAC-seq Analysis

Clean data were aligned to human reference genome hg19 using Bowtie2 (Kodama et al., 2012) by default. MACS2 tool (Feng et al., 2012) was used to call significant peaks. Increased or decreased accessibility of senescence-related ATAC-seq peaks were defined as IARs (increased accessibility regions) or DARs (decreased accessibility regions), respectively. The differential peaks were further used to find enriched TF motifs by script of Homer software (Heinz et al., 2010). GO enrichment analysis of differential peaks were performed using the Genomic Region Enrichment of Annotation Tool, GREAT server (http://great.stanford.edu/public/html/) (McLean et al., 2010). IARs and DARs were input in bed format and set to Grch37 human genome. Results were displayed in bubble plots of the GO biological process term enrichment.

### Visualization of ATAC-seq and ChIP-seq Data

BACH1-ChIP-seq and ATAC-seq visualization were performed by deepTools (Ramírez et al., 2016) and visualized on WashU Epigenome browser (http://epigenomegateway.wustl.edu/browser/). Heatmaps and signal plots were generated by deepTools (3.3.0).

### Immunohistochemistry (IHC)

Immunohistochemistry (IHC) was performed on paraffin-embedded mice aorta sections. Sections were dewaxed by xylene and rehydrated in ethanol solutions of decreasing concentration, permeabilized with 1% Triton X-100 for 20 min and were heated in 95°C water bath for antigen retrieval. Next, sections were incubated with 5% donkey serum for 1 h at room temperature to block nonspecific binding sites. Then sections were incubated with primary antibody anti-BACH1 (14018-1-AP; Proteintech, Rosemont, IL, USA) overnight at 4°C. On the next day, sections were incubated with HRP-conjugated secondary antibodies, subsequently stained with DAB solution (Cwbio, Beijing, China) and counterstained with hematoxylin.

### Cell Culture

Human umbilical vein endothelial cells (HUVECs) were isolated from fresh umbilical cord of normal parturients as described previously (Han et al., 2008). The procedure was approved by the Ethics Committee of Experimental Research at Fudan University. HUVECs were cultured in 5% CO_2_ incubator at 37°C and maintained in endothelial cell medium (ScienCell, Carlsbad, CA, USA) supplemented with 5% fetal bovine serum, 1% endothelial cell growth supplement, and 1% penicillin/streptomycin (Sciencell, Carlsbad, CA, USA). Cells at passages seven to nine were used for experiments. After BACH1 overexpression or knockdown, HUVECs were treated with H_2_O_2_ of different concentrations for 48 h to induce premature senescence.

### RNA Interference and Adenovirus Infection

BACH1 silencing was either implemented *via* small interfering RNA (siRNA) or recombinant adenovirus. Briefly, HUVECs were transfected with the *Bach1* siRNAs (*Bach1*siRNA) or the control siRNAs (ConsiRNA) with Lipofectamine 3000 Transfection Reagent (Invitrogen, Carlsbad, CA, USA) following the instructions of the manufacturer. The transfection medium was replaced by culture medium after 8 h, and the cells were incubated for another 48 h. The sequences of siRNAs are listed in Supplementary Table S1.1. Alternatively, recombinant adenoviruses carrying shRNAs targeting human BACH1 (Ad-sh*Bach1*) (Hanbio, Shanghai, China) were used to knock down BACH1. Adenoviruses Ad-NC were used as control. To overexpress BACH1, the recombinant adenoviruses encoding human BACH1 gene (Ad-BACH1) were used (Hanbio, Shanghai, China). Ad-BACH1 vector is also coded for green fluorescence protein (GFP) expression, and adenoviruses encoding GFP (Ad-GFP) were used as control. HUVECs were seeded in six-well cell culture plates at 2 × 10^5^ cells and were infected with adenovirus to knockdown or overexpress BACH1.

### Western Blotting

Western Blotting was performed as described previously (Wei et al., 2019). Briefly, cells were lysed with RIPA lysis buffer supplemented with protease inhibitors on ice for 30 min, and then centrifuged at 12,000× rpm for 15 min at 4°C. Equal amounts of total proteins were mixed with 6× SDS loading buffer and heated to 100°C for 5 min. Proteins were separated by SDS-PAGE and transferred to polyvinylidene difluoride membranes (Millipore, Billerica, MA, USA). Membranes were then blocked with 5% milk, and proteins were detected using their respective antibodies. Protein bands were visualized by Tanon Imaging System (Tanon Science and Technology Ltd., Shanghai, China). The intensity of the bands was measured *via* densitometric analysis with ImageJ software and normalized to the control. Antibodies to P21 (2947; Cell Signaling Technology, Danvers, MA, USA), P53 (10442-1-AP; Proteintech, Rosemont, IL, USA), BACH1 (sc-271211; Santa Cruz Biotechnology, Santa Cruz, CA, USA), β-ACTIN (20536-1-AP; Proteintech, Rosemont, IL, USA) were used. Each experiment was repeated three times independently.

### Quantitative Real-Time Reverse Transcription-Polymerase Chain Reaction (qRT-PCR)

Quantitative real-time reverse transcription-polymerase chain reaction (qRT-PCR) was performed as described previously (Wei et al., 2019). Total RNA from HUVECs was extracted using TRIzol reagent (Invitrogen, Carlsbad, CA, USA) following the instructions of the manufacturer. mRNA was reverse transcribed to cDNA with ReverTra Ace qPCR RT Kit (FSQ-101; TOYOBO, Osaka, Japan). qRT-PCR was performed using TOROGreen qPCR Master Mix (QST-100; Shanghai, China). Threshold amplification values (Ct) were analyzed using Thermo Real-Time System (Thermo Scientific, Waltham, MA, USA). Transcript quantities were calculated based on Ct values and normalized to the control. Sequences of the primers used were listed in Supplementary Table S1.2. Each experiment was repeated three times independently.

### Chromatin Immunoprecipitation (ChIP)

Chromatin immunoprecipitation (ChIP) was performed as described previously (Wei et al., 2019). Briefly, HUVECs were cross-linked with 1% formaldehyde (F8775; Sigma-Aldrich, St. Louis, MO, USA) for 10 min, terminated by 0.125 M glycine for 5 min at room temperature. The genomic DNA was sheared into 200- to 1,000-base pair lengths by sonication. Then samples were incubated with antibody anti-BACH1 at 4°C overnight, and 1% chromatin was reserved as input DNA. Next day, Protein A/G PLUS-Agarose (sc-2003; Santa Cruz Biotechnology, Santa Cruz, CA, USA) was bound to antibody for 6 h, and the beads were washed. Then the DNA/protein complexes were eluted. Detached DNA was purified with a DNA purification kit (Magen Biotechnology, Guangzhou, China). The immunoprecipitated DNA was analyzed by qRT-PCR and was expressed as percentage of the input DNA. The primer sequences are listed in Supplementary Table S1.2.

### Senescence-Associated β-galactosidase (SA-β-gal) Sstaining

Senescence-associated β-galactosidase (SA-β-gal) staining was performed using a staining kit (Beyotime, Shanghai, China) following the instructions of the manufacturer. Briefly, after H_2_O_2_ treatment, HUVECs were fixed for 15 min. Then cells were incubated with the staining solution mix for 37°C overnight without CO_2_. Positive senescent cells were stained blue. SA-β-gal positive cells were counted under a brightfield microscope.

### Statistical Analysis

Statistical analysis was performed using Prism 8.0 (GraphPad Software, La Jolla, CA, USA). All results are shown as mean ± standard error of the mean (SEM). Comparisons between two groups were determined by unpaired two-tailed Student’s *t*-test. Differences between multiple groups were determined by one-way ANOVA, followed by Tukey *post-hoc* tests. A value of *p* < 0.05 was considered as the criterion of significance.

## Results

### scRNA-seq Analysis of Aortic and Coronary Arteries From Young and Old Monkeys

**FIGURE 1:**
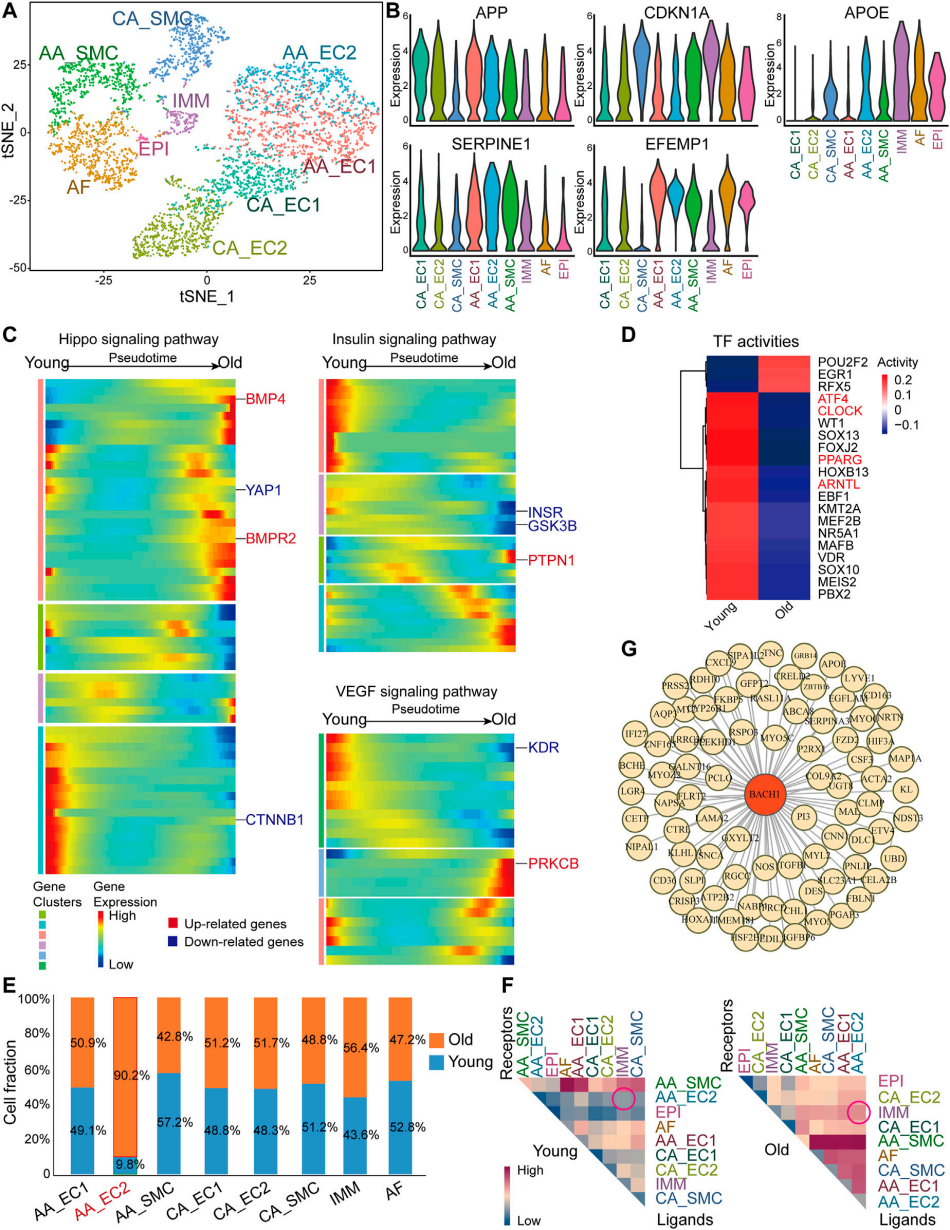
Single-cell RNA sequencingscRNA-seq) analysis of aortas and coronary arteries from young and old monkeys. **(A)** A t-SNE diagram of the global cell population (5,291 cells). The plot is color-coded by cell clusters identified by unsupervised analysis. **(B)** Violin plot of normalized expression of aging-related genes amyloid beta precursor protein (*APP*), *CDKN1A*, apolipoprotein (*APOE*), serpin family E member 1 (*SERPINE1*), and EGF-containing fibulin extracellular matrix protein 1 (*EFEMP1*). **(C)** Pseudotime heatmap showing relative expression of signaling pathway-related genes from the state of young to old in endothelial cells (ECs) from the coronary vasculature (CA_ECs). **(D)** Heatmap of selected TF activities inferred by DoRothEA between young and old monkeys. Red color genes are potential functional regulators of interest. **(E)** Cell composition of ECs from the aortic arches (AA_EC) (AA_EC1), AA_EC2, smooth muscle cells fraction (AA_SMC), ECs from the coronary vasculature (CA_EC1), CA_EC2, CA_SMC, adventitial fibroblast (AF), immune cell (IMM) by age (young/old). Cluster AA_EC2 highlighter in red. **(F)** Heatmap of mean interaction numbers of ligands and receptors between cell clusters. Rows represent ligands, and columns represent receptors. **(G)** Network visualization of BTB and CNC homology 1 (BACH1)-related regulation in EC populations. Yellow nodes represent old/young differentially expressed genes (DEGs) of AA_EC1, AA_EC2, CA_EC1, and CA_EC2. BACH1 highlighted in red.

To investigate the profile of RNA expression changes during primate vascular aging, we analyzed a published scRNA-seq dataset of aortic arches and coronary arteries from young and old monkeys (GEO accession nr. GSE117715) (Zhang et al., 2020). We identified nine clusters of different cell types, including ECs, SMCs, epicardial cells (EPIs), adventitial fibroblasts (AFs), and immune cells (IMMs) (Figure 1A). ECs from the aortic arches (AA_EC) comprise two populations: cluster AA_EC1 is enriched for angiogenesis regulators [bone morphogenetic protein 4 (*BMP4*), TEK receptor tyrosine kinase (*TEK*)] and cluster AA_EC2 expresses inflammatory related genes [interleukin 1 receptor type 1 (*IL1R1*) and interleukin 13 receptor subunit alpha 2 (*IL13RA2*)]. ECs from the coronary vasculature (CA_EC) included two populations: cluster CA_EC1 expresses dedicator of cytokinesis 9 (*DOCK9*) and protein tyrosine phosphatase receptor type B (*PTPRB*); cluster CA_EC2 is enriched for fatty acid-binding protein 4 (*FABP4*) and plasmalemma vesicle-associated protein (*PLVAP*). We observed that aging-related genes, including amyloid beta precursor protein (*APP*), *CDKN1A*, apolipoprotein (*APOE*), serpin family E member 1 (*SERPINE1*), and EGF containing fibulin extracellular matrix protein 1 (*EFEMP1*), were highly expressed within cells from aortic and coronary arteries (Figure 1B and Supplementary Figure S1). Coronary arteries are responsible for heart perfusion and homeostasis. Hippo, insulin, and VEGF signaling pathways are crucial for maintaining vascular homeostasis (Kubota et al., 2011; He et al., 2018; Grunewald et al., 2021). Therefore, we investigated the changes of the genes in these signaling pathways in ECs of coronary arteries. We analyzed the old/young (O/Y) DEGs in ECs of coronary arteries population and performed pseudotime analysis of this population in monkey (Figure 1C). Hippo signaling pathway features upregulated fibrosis-related genes [*BMP4* and bone morphogenetic protein receptor type 2 (*BMPR2*)] and downregulated proliferation-related genes [Yes1 associated transcriptional regulator (*YAP1*) and catenin beta-1 (*CTNNB1*)] toward old cell state (Figure 1C). Most insulin signaling pathway genes [(insulin receptor (*INSR*) and glycogen synthase kinase 3 beta (*GSK3B*)] were downregulated when the cells were steered toward old cell state, while protein tyrosine phosphatase non-receptor type 1 (*PTPN1*), a key negative regulator of insulin signaling was upregulated (Figure 1C). Analysis of the VEGF signaling pathway demonstrated that VEGF receptor 2 (*KDR*) was decreased, while endothelial dysfunction-related gene protein kinase C beta (*PRKCB*) was increased toward old cell state (Figure 1C). These results suggest that the genetic signature of aged ECs of coronary arteries from monkey are associated with decrease in angiogenesis and increase in endothelial dysfunction. TF activity analysis of aortic arches and coronary arteries from young and old monkeys revealed low transcriptional activity of circadian rhythms related TFs [clock circadian regulator (*CLOCK*), aryl hydrocarbon receptor nuclear translocator like (*ARNTL*), and peroxisome proliferator activated receptor gamma (*PPARG*)] and endoplasmic reticulum stress-related TF-activating transcription factor 4 (*ATF4*) in old vasculature (Figure 1D). Next, we explored whether the proportion of cell types was altered during primate vascular aging. Among the identified cell types, there was a significant increase in aortic arches endothelial cells cluster 2 (AA_EC2) and a slight decrease in smooth muscle cells fraction (AA_SMC) in old arteries (Figure 1E). Moreover, immune cells (IMM) were slightly increased in vessels of old monkeys (Figure 1E). Other cells showed similar fraction in arteries from young and old monkeys. Intercellular communication between heterogenous populations is important for aging process (Tang et al., 2020). We then analyzed the interaction of various vascular cell types in young and aged vessels. We observed an increased interaction of AA_EC2 and other cell types, especially IMM, in aged vessels (Figure 1F). To identify regulators related to vascular aging, we constructed gene regulatory networks of EC-associated aging genes and transcription factors with GENIE3. We identified BACH1 as a master regulator of aging-related genes in both coronary and aorta ECs of monkey (Figure 1G).

### scRNA-seq Analysis of Young and Old Mouse Heart endothelial cell

**FIGURE 2:**
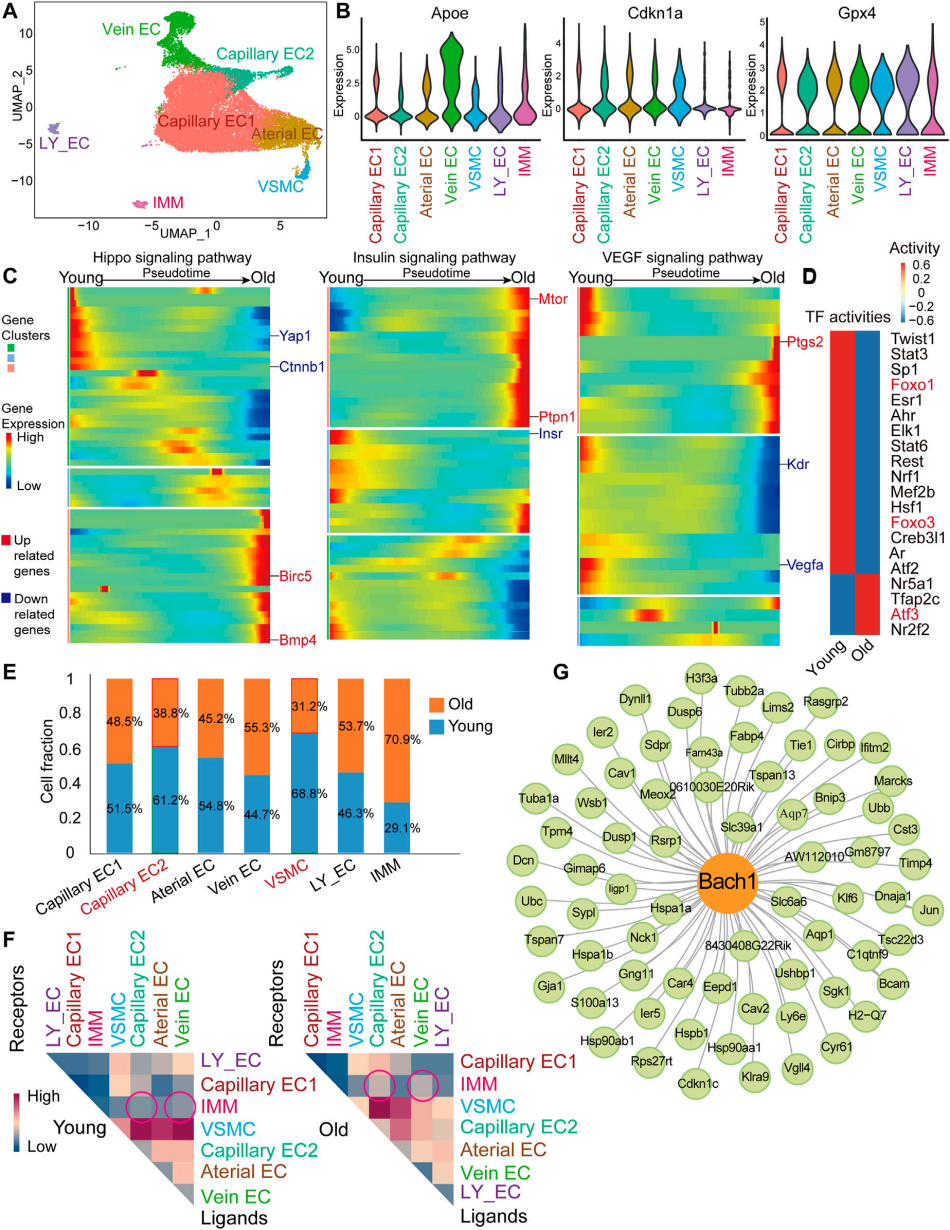
scRNA-seq analysis of young and old mouse heart ECs. **(A)** UMAP plot of 26,385 cells for the mouse global cell population. EC, lymphatic EC-like cells (LY_EC), vascular smooth muscle cell (VSMC), and IMM. **(B)** Violin plot of normalized expression of aging-related genes, Apoe, Cdkn1a, and Gpx4. **(C)** Pseudotime heatmap showing relative expression of signaling pathway-related genes from the state of young to old in Capillary EC2. **(D)** Heatmap of selected TF activities inferred by DoRothEA between young and old mice. Red color genes are transcription factors (TFs) of interest. **(E)** Cell composition of seven Seurat clusters by age (young/old). **(F)** Heatmap of mean interaction numbers of ligands and receptors between cell types. Rows represent ligands, and columns represent receptors. **(G)** Network visualization of Bach1-related regulation in EC clusters. Green nodes represent old/young DEGs of Capillary EC1, Capillary EC2, Arterial EC, and Vein EC. Bach1 shown as the yellow node.

Many of scRNA-seq studies so far are available from aging modeling in mice, and thus, it is relevant to define the cellular and molecular properties of aged cardiac vasculature in monkeys also in respect to mice. We analyzed a published scRNA-seq dataset of cardiac vasculature (including coronary arteries, coronary veins, and capillaries) from young and old mice (GEO accession nr. GSE163822) (Figure 2A) (Emechebe et al., 2021). We identified seven clusters in the young and aged cardiac endothelium. The EC populations identified included arterial ECs (Jag1^+^ and Gja4^+^), venous ECs (Nr2f2^+^ and Vwf^+^), two capillary EC clusters including capillary EC1 (Cxcl12^+^) and capillary EC2 (Cdk1^+^ and E2f1^+^), and lymphatic ECs (Lyve1^+^). Other populations included SMCs (Tagln^+^ and Myh11^+^) and immune cells (IMMs, Ptprc^+^) (Figure 2A). Aging-related genes *Apoe* and *Cdkn1a* (encoding for P21) were highly expressed in mouse cardiac ECs (Figure 2B and Supplementary Figure S2A), similar with that of monkey coronary arteries. Besides, the antioxidant peroxidase glutathione peroxidase 4 (*Gpx4*) was widely expressed in mouse cardiac ECs (Figure 2B). Capillary rarefaction and dysfunction are associated with vascular aging (Das et al., 2018; Grunewald et al., 2021), we then performed monocle analysis on capillary ECs and observed an aged-responsive trajectory within proliferation-related CA-EC2 cells (Supplementary Figure S2B). Also, the fraction of proliferation-related Capillary EC2 cluster declined (Figure 2E). Therefore, we proceeded with monocle analysis on Capillary EC2 (Figure 2C). We found that *Yap1* and *Ctnnb1* decreased, while fibrosis-related genes *Bmp4* and baculoviral IAP repeat containing 5 (*Birc5*) increased toward old cell state in the Hippo pathway (Figure 2C). Also, *Insr* decreased, while *Ptpn1* and mechanistic target of rapamycin kinase (*Mtor*) increased toward old cell state in insulin pathway (Figure 2C). Finally, *Kdr* and vascular endothelial growth factor A (*Vegfa*) decreased, while inflammatory response-related gene prostaglandin-endoperoxide synthase 2 (*Ptgs2*) increased toward old cell state in VEGF signaling pathway (Figure 2C). These results indicate that aged Capillary EC2 was characterized by low proliferative capacity, insulin resistance and inflammation, which may contribute to low blood perfusion in aged heart. Forkhead box O3 (*Foxo3*) and forkhead box O1 (*Foxo1*) are protective factors against vascular aging (Zhang et al., 2020). TF activity analysis revealed their low transcriptional activity in old vasculature (Figure 2D). In contrast, activating transcription factor 3 (*Atf3*) activity was high in aged vessels, which has been shown to promote cellular senescence (Zhang et al., 2021). We then detected the proportion of cell types in aged cardiac vessels of mouse. There was a slight decrease of smooth muscle cells in the heart of old mouse, similarly in the aortas of old monkey (Figure 2E). Moreover, we explored the interaction of various vascular cell types in aged cardiac vessels of mouse. We observed slightly increased interactions between IMM and Vein EC or Capillary EC2, as well as a decreased self-interaction of vein ECs in aged vessels (Figure 2F). Additionally, gene regulatory networks analysis identified BACH1 as a master regulator of aging-related genes in cardiac ECs of mouse (Figure 2G), similarly to the finding in the monkey dataset (Figure 1G).

### ATAC-seq Analysis of Young and Senescent endothelial cells

**FIGURE 3:**
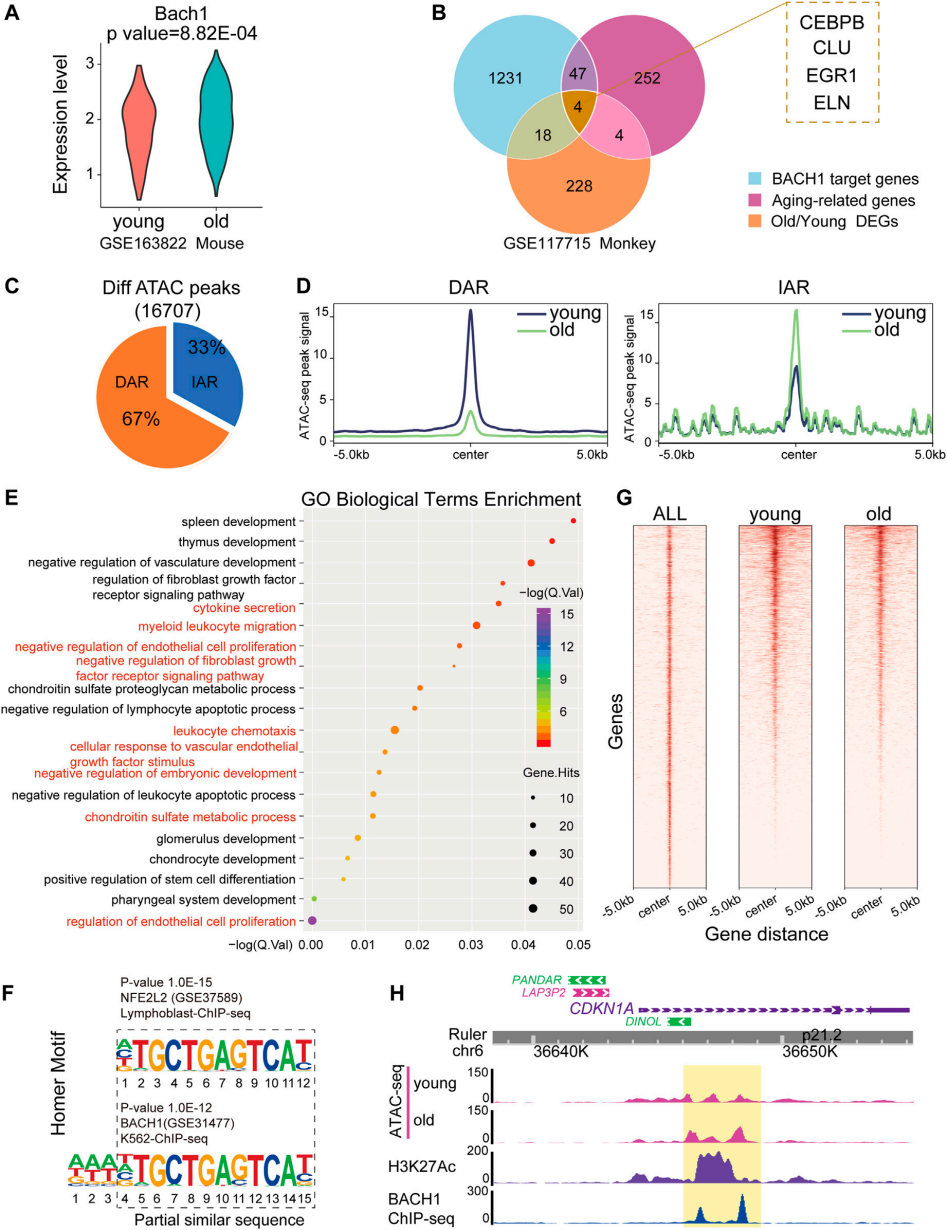
ATAC-seq analysis of young and senescent ECs. **(A)** Violin plot showing normalized expression of BACH1 in mouse ECs. **(B)** Venn diagram showing overlapping of 1) old/young monkey DEGs; 2) aging-related genes from GenAge database, and 3) target genes of BACH1 from ChEA3 and TRRUST resources. **(C)** Pie chart of differential ATAC-seq peaks identified by MACS2. During senescence, the accessibility of senescence-related peaks increased or decreased is defined as increased accessibility regions (IARs) or decreased accessibility regions (DARs). **(D)** The ATAC-seq signals of young samples in IARs and DARs are different from that of old samples (region center ±5 kb). **(E)** GO enrichment analysis of IARs performed on the GREAT server. The aging and endothelial-related terms are highlighted in red. **(F)** The known transcription factors’ motifs of differential peaks of ATAC-seq. **(G)** Heatmap of BACH1-binding profiles and ATAC-seq of young and old at BACH1-enriched regions (region center ±5 kb). **(H)** Representative snapshots of ATAC-seq of young and old samples, ChIP-seq of H3K27Ac and BACH1 tracks on the enhancer and promoter neighboring *CDKN1A*.

To detect whether the expression of BACH1 is associated with aging-related genes, we analyzed the expression of BACH1 in the vasculature of mice and found that the expression of BACH1 was upregulated in cardiac ECs of old mouse (Figure 3A). We compared aging-related genes with BACH1 target genes and O/Y DEGs in monkey. Fifty-one aging-related genes were BACH1 target genes in the vessels of monkey, including CCAAT enhancer-binding protein beta (*CEBPB*), clusterin (*CLU*), and early growth response 1 (*EGR1*) (Figure 3B). To further explore the mechanisms of vascular senescence, we analyzed a published transposase-accessible chromatin with high-throughput sequencing (ATAC-seq) dataset from young and senescent human umbilical vein endothelial cells (HUVECs) (GEO accession nr. GSE157867) (Zhang et al., 2021). Increased or decreased accessibility of senescence-related ATAC-seq peaks were defined as IARs (increased accessibility regions) or DARs (decreased accessibility regions), respectively. We called different regions from young and senescent HUVECs (Figure 3C). Analysis of the signal profiles of ATAC-seq data revealed that chromatin accessibility of old HUVECs declined at the regions of decreased accessibility and enhanced at the regions of increased accessibility compared with those of young HUVECs (Figure 3D). GO analysis of increased accessibility regions indicates enrichment in negative regulation of endothelial cell proliferation, cellular response to vascular endothelial growth factor stimulus, and myeloid leukocyte migration (Figure 3E). Motif analysis of different opening of ATAC-seq peaks revealed that the motif of BACH1, similar to the motif of NFE2, such as bZIP transcription factor 2 (NFE2L2), was enriched in the accessible chromatins (Figure 3F). Heatmaps showed significant colocalization of BACH1 at regions of open chromatin in both young and senescent HUVECs (Figure 3G). Representative snapshots of ChIP-seq (GEO accession nr. GSM935580) tracks indicate that BACH1 was enriched in the enhancers of *CDKN1A* (encoding for P21) gene, and these regions were more accessible in senescent HUVECs (Figure 3H). Together, these results suggest that BACH1 may be an important transcription factor for senescence regulation in ECs.

### BACH1 Is Upregulated in the Aorta of Old Mice, Binds to *CDKN1A* gene Enhancer, and Activates Its Transcription

**FIGURE 4:**
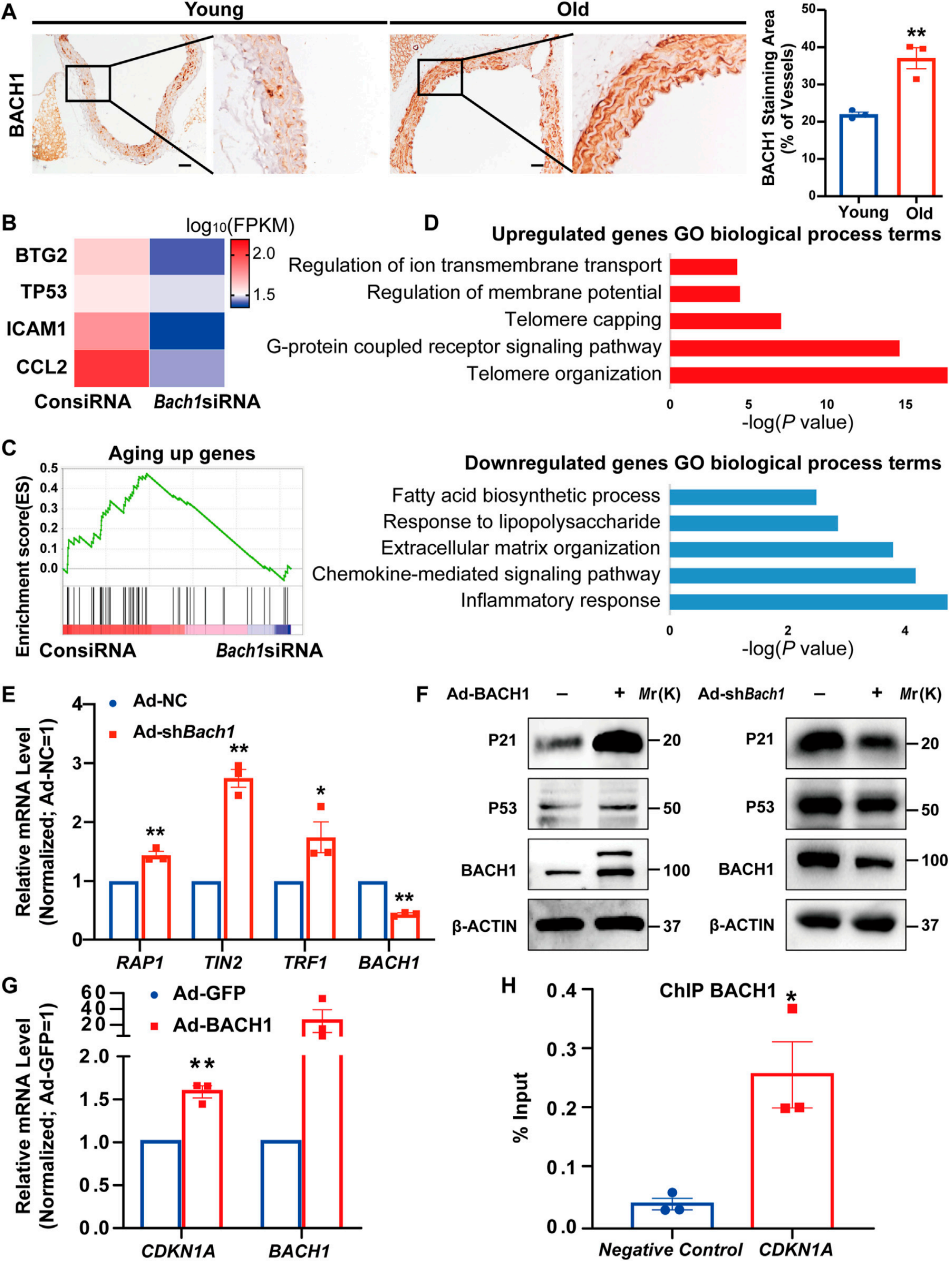
BACH1 is upregulated in the aorta of old mice, and binds to the *CDKN1A* gene enhancer and activates its transcription. **(A)** Representative images of immunohistochemical analysis for BACH1 staining in the aorta of young mice and old mice (*n* = 3 per group). Scale bar, 50 μm. (*n* = 3 independent experiments, data are mean ± SEM, unpaired 2-tailed *t*-test, *p < 0.05, **p < 0.01). **(B)** Heatmap illustrating RNA expression of selected genes related to aging in human umbilical vein endothelial cells (HUVECs) transfected with ConsiRNA or *Bach1*siRNA. **(C)** Gene set enrichment analysis (GSEA) showing genes repressed by BACH1 deletion were associated with aging. **(D)** The Gene Ontology analysis of the significantly downregulated or upregulated genes in BACH1 silencing HUVECs compared with the control HUVECs based on the RNA-seq data. **(E)** qRT-PCR analysis of TERF2-interacting protein (*RAP1*), TERF1-interacting nuclear factor 2 (*TIN2*), telomeric repeat binding factor 1 (*TRF1*), and *BACH1* mRNA levels in HUVECs after BACH1 knockdown *via* adenovirus infection (*n* = 3 independent experiments, quantitative analysis was normalized to the Ad-NC group, data are mean ± SEM, unpaired 2-tailed *t*-test, *p < 0.05, **p < 0.01). **(F)** Western blot analysis of P21 and P53 and BACH1 protein levels in HUVECs after BACH1 overexpression or knockdown *via* adenovirus infection. **(G)** The mRNA expression of CDKN1A and BACH1 in Ad-BACH1-HUVECs and Ad-GFP-HUVECs (*n* = 3 independent experiments, quantitative analysis was normalized to the Ad-GFP group, data are mean ± SEM, unpaired 2-tailed *t*-test, **p < 0.01). **(H)** Chromatin immunoprecipitation (ChIP)-polymerase chain reaction (qPCR) analysis validates the enrichment of BACH1 at the enhancer of *CDKN1A* gene (*n* = 3 independent experiments, data are mean ± SEM, unpaired 2-tailed *t*-test, *p < 0.05).

To determine whether the expression of BACH1 was altered in aged vessels, we analyzed the expression and localization of BACH1 in aortas from young and old mice by immunostaining. The expression of BACH1 in the intima, media, and adventitia of mouse aortas from old mice was higher than that of young mice (Figure 4A), suggesting the involvement of BACH1 in vascular aging. To identify potential mechanisms underlying BACH1-related vascular aging, we conducted whole-genome RNA deep sequencing to profile the transcriptomes of HUVECs transfected with *Bach1* siRNAs (*Bach1*siRNA) or the control siRNAs (ConsiRNA). The downregulation of BACH1 in *Bach1*siRNA-transfected HUVECs was confirmed by qRT-PCR (Supplementary Figure S3). We observed the downregulation of aging-related genes, including *TP53*, *CCL2* (*MCP-1*), *ICAM1*, and *BTG2* in *Bach1*siRNA-HUVECs than in ConsiRNA-HUVECs (Figure 4B). Gene set enrichment analysis (GSEA) also showed that the aging-related genes were significantly downregulated in the *Bach1*siRNA-HUVECs group (Figure 4C). Many of the upregulated genes were associated with telomere organization, while many of the downregulated genes were associated with inflammatory response, which are related to vascular aging (Figure 4D). BACH1 knockdown was associated with significant increases in the expression of telomere-related genes, such as TERF2-interacting protein (*RAP1*), TERF1-interacting nuclear factor 2 (*TIN2*), and telomeric repeat binding factor 1 (*TRF1*) (Figure 4E), which are involved in the regulation of telomere length and protection as a component of the shelterin complex (Grolimund et al., 2013). Consistent with the RNA sequencing data, P21 and/or P53 protein expression was higher in BACH1-overexpressing HUVECs than in the control (Figure 4F), and mRNA level was confirmed by qRT-PCR (Figure 4G). In contrast, BACH1 knockdown decreased the level of P21 and P53 (Figure 4F). Furthermore, ChIP-qPCR confirmed the chromatin occupancies of BACH1 near the enhancer of the *CDKN1A* gene (Figure 4H). These data indicate that BACH1 binds to *CDKN1A* enhancer and activates its transcription.

### BACH1 Aggravates Endothelial Cell senescence Under Oxidative Stress

**FIGURE 5:**
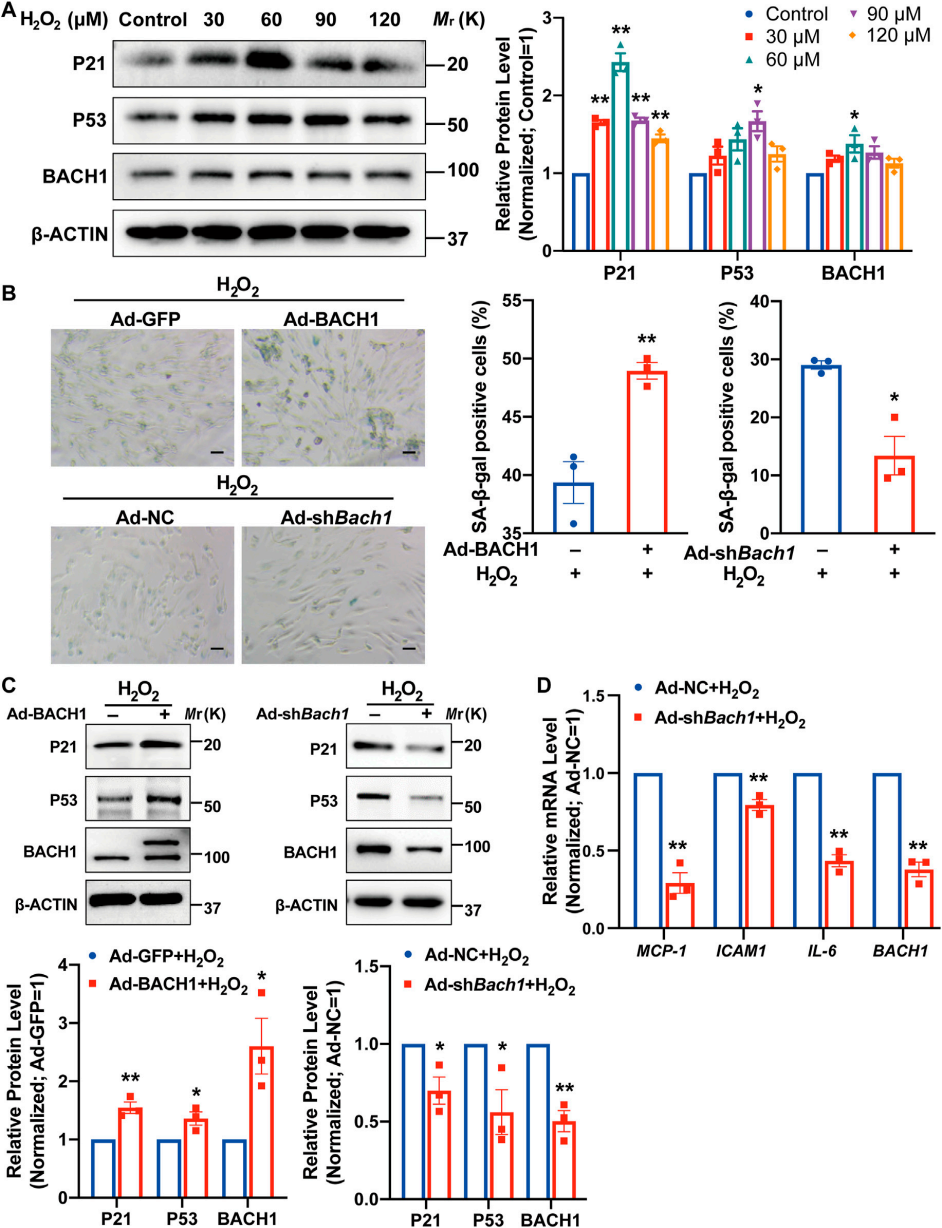
BACH1 aggravates endothelial cell senescence under oxidative stress. **(A)** Western blot analysis of P21, P53, and BACH1 protein levels in HUVECs under H_2_O_2_ (0, 30, 60, 90, 120 μM) treatment for 12 h. Quantitative analysis was normalized to control (*n* = 3 independent experiments, data are mean ± SEM. One-way ANOVA followed by Tukey *post-hoc* tests, *p < 0.05 versus Control, **p < 0.01 versus Control). **(B)** Representative images of SA-β-gal staining for HUVECs under H_2_O_2_ (60 or 120 μM) treatment for 12 h after BACH1 overexpression or knockdown *via* adenovirus infection. Scale bar, 50 μm. Quantitative analysis was measured by the percentage of SA-β-gal positive cells (*n* = 3 independent experiments, data are mean ± SEM, unpaired 2-tailed *t*-test, *p < 0.05, **p < 0.01). **(C)** Western blot analysis of P21 and P53 and BACH1 protein levels in HUVECs under H_2_O_2_ treatment for 12 h after BACH1 overexpression or knockdown *via* adenovirus infection. Quantitative analysis was normalized to the control group (*n* = 3 independent experiments, data are mean ± SEM, unpaired 2-tailed *t*-test, *p < 0.05, **p < 0.01). **(D)** qRT-PCR analysis of *MCP-1*, *ICAM1*, and *IL-6* mRNA levels in HUVECs under H_2_O_2_ treatment for 12 h after BACH1 knockdown *via* adenovirus infection. Quantitative analysis was normalized to the control group (*n* = 3 independent experiments, data are mean ± SEM, unpaired 2-tailed *t*-test, *p < 0.05, **p < 0.01).

We then determined whether BACH1 affects endothelial cell senescence under oxidative stress. BACH1 expression was upregulated after H_2_O_2_ stimulus for 12 h and peaked at the concentration of 60 μM (Figure 5A). Simultaneously, the classic senescence pathway P53/P21 was activated under oxidative stress (Figure 5A). Staining of senescence-associated β-galactosidase, a marker for cellular senescence, showed that BACH1 knockdown significantly attenuated endothelial cell senescence under H_2_O_2_ treatment, while BACH1 overexpression aggravated H_2_O_2_-induced senescence in HUVECs (Figure 5B). Consistently, overexpression of BACH1 increased the protein level of P53 and P21 under H_2_O_2_ treatment, and BACH1 knockdown decreased these protein levels (Figure 5C). Furthermore, qRT-PCR assays showed that knockdown of BACH1 reduced the expression of senescence-associated secretory phenotype (SASP) genes (*MCP-1*, *ICAM1*, and *IL-6*) upon H_2_O_2_ treatment (Figure 5D). Overall, these results suggest that BACH1 aggravates endothelial cell senescence under oxidative stress.

## Discussion

In the present study, we revealed an increased interaction of ECs and immunocytes in aged vasculature of both monkeys and mice, whereas proliferation-related cardiac capillary ECs were significantly decreased in the heart of aged mouse. We identified BACH1 as a master regulator of endothelial senescence during aging. BACH1 was upregulated in the aorta and cardiac ECs of old mice. BACH1 also aggravated endothelial cell senescence under oxidative stress *via* binding to *CDKN1A* gene enhancer and activating its transcription. Our findings provide crucial insights into the regulatory role of BACH1 in vascular aging.

Recent studies established single-cell transcriptomic atlas of aortic and coronary arteries in aged monkey and heart ECs in aged mouse (Zhang et al., 2020; Emechebe et al., 2021), providing rich resources for the analysis of cellular heterogeneity in aged vessels from monkey and mouse. However, cell communication between heterogenous populations, the proportion of different cell types, and the transcriptional factor activity in young and aged vessels from monkey and mouse are still unknown. Here, we observed a significantly decreased subpopulation of heart capillary ECs in aged mouse. This class of capillary ECs highly expressed cell cycle-related genes including cyclin-dependent kinase 1 (*Cdk1*) and E2F transcription factor 1 (*E2f1*). The decrease in this EC population in the aged mice heart may be associated with decreased angiogenesis during vascular aging. Moreover, we found one of the aortic arches EC clusters (AA_EC2) enriched in old monkey ECs, indicating an aging-specific EC state. This increased aorta EC subpopulation in old monkey is related to vascular inflammation. Chronic, sterile, low-grade inflammation is a hallmark of the aging process (Ungvari et al., 2018). The increased proinflammatory ECs may induce inflammatory cytokine release, which results in proinflammatory microenvironment in the vascular wall and promotes vascular dysfunction and atherosclerosis in aging. Non-myocytes (endothelial cells, fibroblasts, and immune cells) have been shown to regulate the senescence of cardiomyocytes in the local microenvironment and contribute to cardiac aging (Tang et al., 2020). Similarly, there is a slight decline in SMCs in both old monkey and old mouse, and the loss of SMCs may lead to impaired vasoconstriction and vessel wall thinning, both contributing to vascular aging. In this study, we discovered an increased interaction of arterial ECs and immunocytes, as well as a decreased self-interaction of vein ECs in aging, which may be associated with loosened intercellular junctions and increased vascular permeability. Further studies are required to explore how the changes in cellular interaction contributes to vascular aging.

The Hippo pathway is important for angiogenesis and cardiovascular homeostasis (He et al., 2018; Azad et al., 2019). We found that the fibrosis-related genes were upregulated and the proliferation-related genes were downregulated in Hippo pathway in both aged monkey and mouse cardiac vessels. The imbalance of Hippo pathway in aging vessels may lead to the decrease in angiogenesis and low blood perfusion. Previous studies discovered that aging-related deterioration of vascular function is due to VEGF signaling insufficiency (Grunewald et al., 2021). In line with the previous studies, we found that VEGF receptor 2 was decreased in both aged monkey and mouse cardiac vessels. Vascular aging is associated with the loss of capillaries, which is partly due to insulin resistance in ECs (Kubota et al., 2011). We found that insulin receptor was decreased in old cardiac ECs of both monkey and mouse. The decrease in insulin receptor may induce insulin resistance and reduction of capillaries in aged ECs. These changes in pathways might explain the decline in angiogenesis and vascular dysfunction during aging. Moreover, circadian is an important factor for aging (Acosta-Rodríguez et al., 2021). Of note, mice have different rhythm behavior from humans, which highlights the need for murine–primate comparison in circadian regulations. This study looked into monkey aortas and coronary arteries and mouse heart vessels, respectively, but a more systemic, well-defined comparison between mouse and monkey on the same set of vessels is desired, ideally *via* scRNA-seq.

Epigenetic alterations in the key regulators are closely associated with gene expression, contributing to vascular aging (Booth and Brunet, 2016; Zhang W. et al., 2018). Accessible chromatin is strongly associated with active gene regulatory regions (Carter and Zhao, 2021). However, the global change in epigenome in vascular aging remains largely unknown, and a few studies focused on the changes of chromatin accessibility during aging. In this study, we found that BACH1 is located at the region of accessible chromatin in senescent HUVECs, indicating that BACH1 may be important for chromatin accessibility regulation and gene expression in vascular aging. We observed increased chromatin accessibility at BACH1-binding sites of *CDKN1A* enhancer in old HUVECs than in young HUVECs, and our ChIP-qPCR results confirmed the enrichment of BACH1 on *CDKN1A* gene, suggesting that BACH1 might upregulate *CDKN1A* (P21) expression *via* binding to *CDKN1A* enhancer in ECs. A previous study has shown that BACH1 exhibited increased nuclear accumulation following H_2_O_2_ treatment in mouse embryonic fibroblasts in a model of accelerated aging (Pomatto et al., 2019). Our recent study showed that BACH1 recruited an essential pluripotency regulator NANOG and the MLL/SET1 complexes to maintain the activity of promoters and (super-) enhancers in embryonic stem cells (Niu et al., 2021). BACH1 might regulate the enhancer activity and the chromatin accessibility of the *CDKN1A* gene by influencing histone modification.

We found that BACH1 expression was increased both in aortas of aged monkey and cardiac ECs of aged mouse. Many BACH1 target genes are associated with aging or senescence. From scRNA-seq of mouse and monkey aging vasculature, we identified the transcriptional regulatory network of BACH1 and found that BACH1 might regulate extracellular matrix and inflammation in aged vessels. Shelterin complex allows cells to distinguish telomeres from sites of DNA damage and prevent senescence (de Lange, 2005; Lim and Cech, 2021). We found that BACH1 knockdown upregulated three important subunits (RAP1, TIN2, TRF1) of shelterin complex. Knockdown of BACH1 might prevent cellular senescence partly through distinguishing telomeres from sites of DNA damage and maintaining telomere length.

Cellular senescence is defined by the change in cell state to permanent cell cycle arrest (Warboys et al., 2014). Senescence is generally regulated by either P53/P21 or P16/retinoblastoma protein (RB) tumor suppressor pathways (Erusalimsky, 2009). Activation of the P53/P21 pathway is primarily triggered by telomere dysfunction and DNA damage (Donato et al., 2018). P21, a cyclin-dependent kinase inhibitor, is responsible for the initial cell cycle arrest at G1/S or G2/M (Chen et al., 2021). In contrast to apoptosis, senescent cells are stably viable and have the potential to produce or release a wide range of inflammatory cytokines and chemokines, termed SASP (Ungvari et al., 2018). SASP exerts a paracrine function that propagates the senescent phenotype to the neighboring cells (Alique et al., 2020). It also reinforces the cell cycle arrest in an autocrine fashion (Donato et al., 2018). We observed that knockdown of BACH1 downregulated the expression of SASP components and reduced EC senescence induced by oxidative stress. We speculate the protection was through the downregulation of P21 and P53, thus, preventing cell cycle arrest. BACH1 inhibition in EC also prevents mitochondrial ROS production by activating Nrf2 signaling pathway and inducing antioxidant genes, which inhibits the onset of senescence in cells subjected to oxidative stress (Ungvari et al., 2019; Arefin et al., 2020). In addition, BACH1 deficiency was associated with premature senescence in murine embryonic fibroblasts under conditions of oxidative stress (Dohi et al., 2008). The differences in senescence between fibroblasts and ECs remain unexplained, but it is potentially due to differences in cell types, cell state, cellular response to oxidative stress or experimental conditions. Previous report found that BACH1 deficiency did not influence the normal life course of mice (Ota et al., 2014). One possible reason is that the genes induced or reduced during aging in mice may compensate for the effect of BACH1 deficiency. However, whether BACH1 affects vascular aging under pathological condition remains to be investigated.

Taken together, the results presented here show that EC interaction with immune cells appears to be crucial in both aged cardiac vessels of monkey and mouse. BACH1 is a master regulator of EC senescence and vascular aging. Inhibiting BACH1 may serve as a potential therapeutic strategy for vascular aging.

## Data Availability

Cardiovascular and heart aging-related scRNA-seq datasets were obtained from GEO (GSE117715 for crab-eating macaque, GSE163822 for mouse). Count files were integrated separately as Seurat objects for further analysis. ATAC-seq data on HUVEC senescence (GSE157867) were analyzed to uncover the potential regulatory mechanisms between chromatin accessibility and gene expression. ChIP-seq data (GSM935580) were visualized in BACH1-binding heatmaps and signal plots. Transcription factor enrichment analysis *via* orthogonal omics integration provides a large number of target gene sets. We collected 1,300 target genes of BACH1 from ChEA3 (https://amp.pharm.mssm.edu/ChEA3) (Keenan et al., 2019) and TRRUST (www.grnpedia.org/trrust) (Han et al., 2018).
