# Supplementary material for: Single-Cell Analysis Identify Transcription Factor BACH1 as a Master Regulator Gene in Vascular Cells During Aging
Source: Front Cell Dev Biol. 2021 Dec 24;9:786496. doi: 10.3389/fcell.2021.786496 (PMC8740196; doi:10.3389/fcell.2021.786496)
Supplement: Supplementary file 2 [file DataSheet2.docx]

We used publicly available datasets in GSE117715 for aortas and coronary arteries from young and old monkeys scRNA-seq analysis (<https://www.ncbi.nlm.nih.gov/geo/query/acc.cgi?acc=GSE117715>) and the data we download from <https://www.ncbi.nlm.nih.gov/geo/download/?acc=GSE117715&format=file>.

We used publicly available datasets in GSE163822 (https://www.ncbi.nlm.nih.gov/geo/query/acc.cgi?acc=GSE163822) for young and old mouse heart ECs scRNA-seq analysis and the data we download from <https://www.ncbi.nlm.nih.gov/geo/download/?acc=GSE163822&format=file>. (young: GSM4987951_Young_Cardiac_set1.csv, GSM4987952_Young_Cardiac_set2.csv, GSM4987953_Young_Cardiac_set3.csv) (old: GSM4987954_Aged_Cardiac_set1.csv, GSM4987955_Aged_Cardiac_set2.csv, GSM4987956_Aged_Cardiac_set3.csv).

We used publicly available datasets in GSE157867 (https://www.ncbi.nlm.nih.gov/geo/query/acc.cgi?acc=GSE157867) for ATAC-seq analysis during HUVEC senescence and the data we download from <https://www.ncbi.nlm.nih.gov/geo/download/?acc=GSE157867&format=file>

We used publicly available datasets in GSM935580 (https://www.ncbi.nlm.nih.gov/geo/query/acc.cgi?acc=GSM935580) for ChIP-seq analysis during HUVEC senescence and the data we download from https://www.ncbi.nlm.nih.gov/geo/download/?acc=GSM935580&format=file&file=GSM935580%5Fhg19%5FwgEncodeSydhTfbsH1hescBach1sc14700IggrabSig%2EbigWig

Our RNA-seq of Bach1siRNA or ConsiRNA were uploaded to GEO (GEO: GSE185210) https://www.ncbi.nlm.nih.gov/geo/query/acc.cgi?acc= GSE185210

The GEO token to allow review of record GSE185210: mdereqyidbylzez
