# Supplementary material for: Single-Cell Analysis Identify Transcription Factor BACH1 as a Master Regulator Gene in Vascular Cells During Aging
Source: Front Cell Dev Biol. 2021 Dec 24;9:786496. doi: 10.3389/fcell.2021.786496 (PMC8740196; doi:10.3389/fcell.2021.786496)
Supplement: Supplementary file 3 [file DataSheet1.DOCX]

**Supplementary Figure and Figure legend**

**
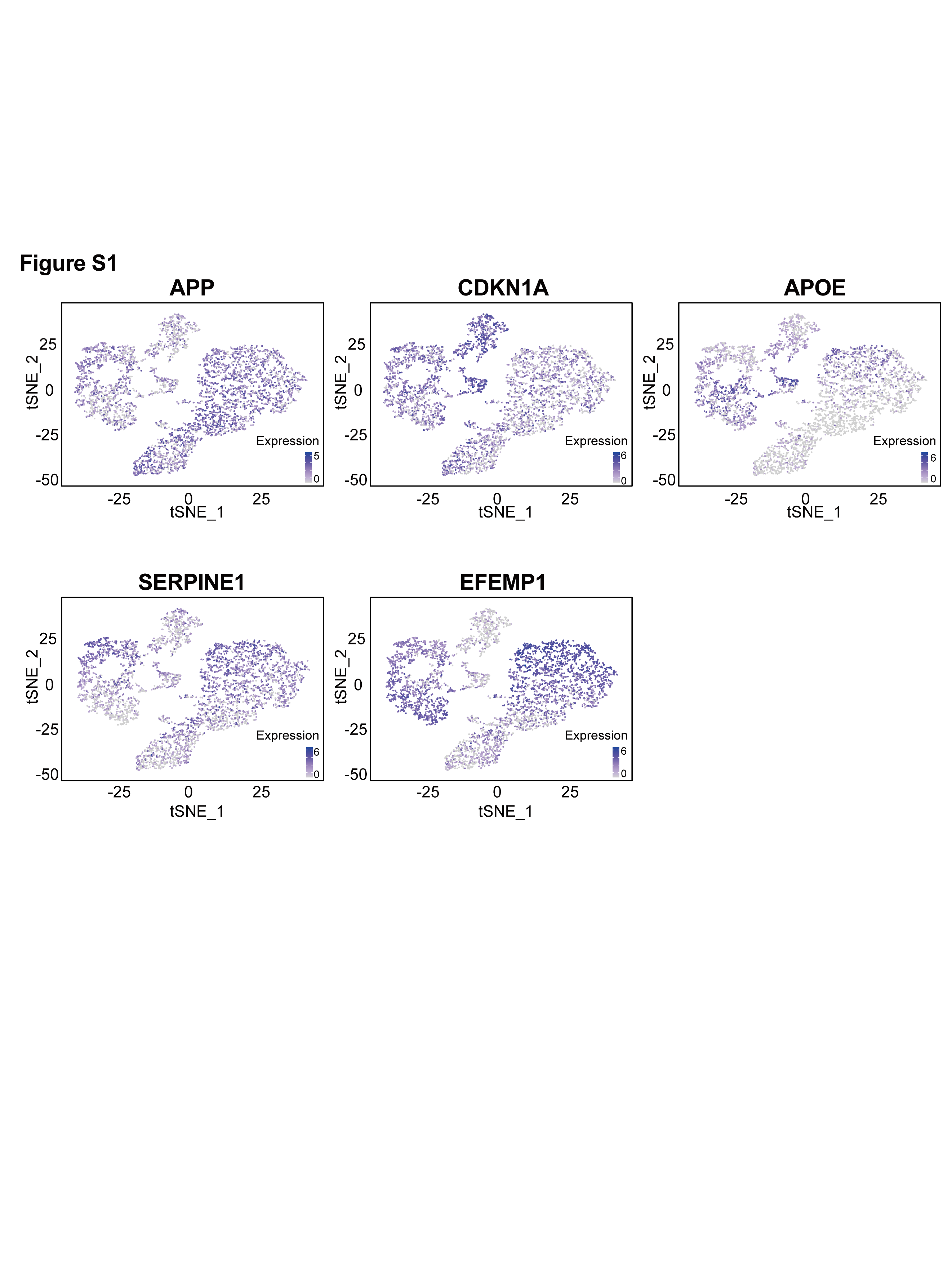
**

**Figure S1. t-SNE representation of the normalized expression of aging-related genes, *APP*, *CDKN1A*, *APOE*, *SERPINE1* and *EFEMP1*.**

**
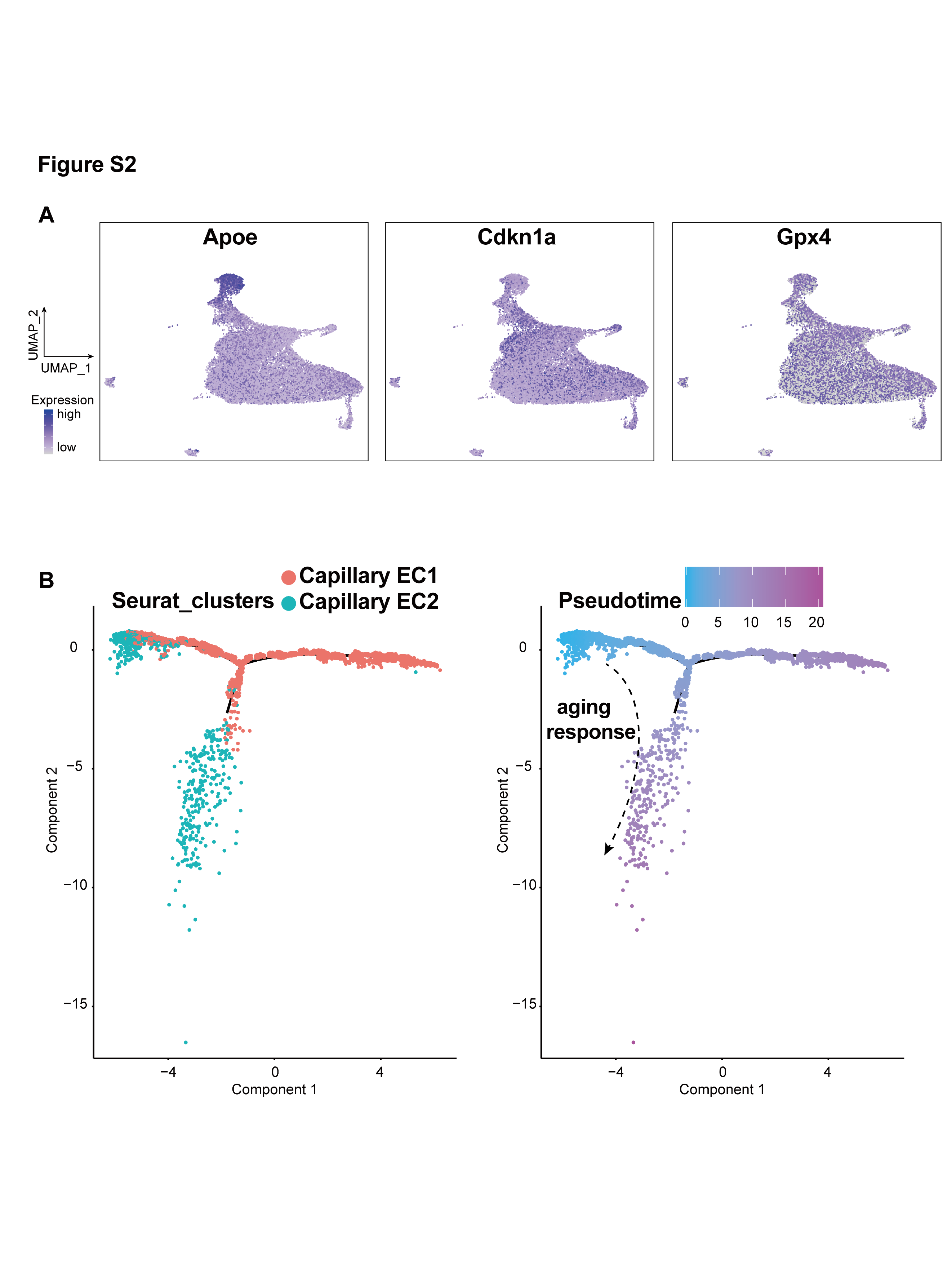
**

**Figure S2. UMAP representation of aging-related genes and pseudotime heatmap analysis.**

1. UMAP representation of the normalized expression of aging-related genes, *Apoe*, *Cdkn1a* (also known as P21) and *Gpx4*.
2. Pseudotime heatmap showing the clusters and pseudotime states from the state of young to old in CAECs, by Monocle2.

**
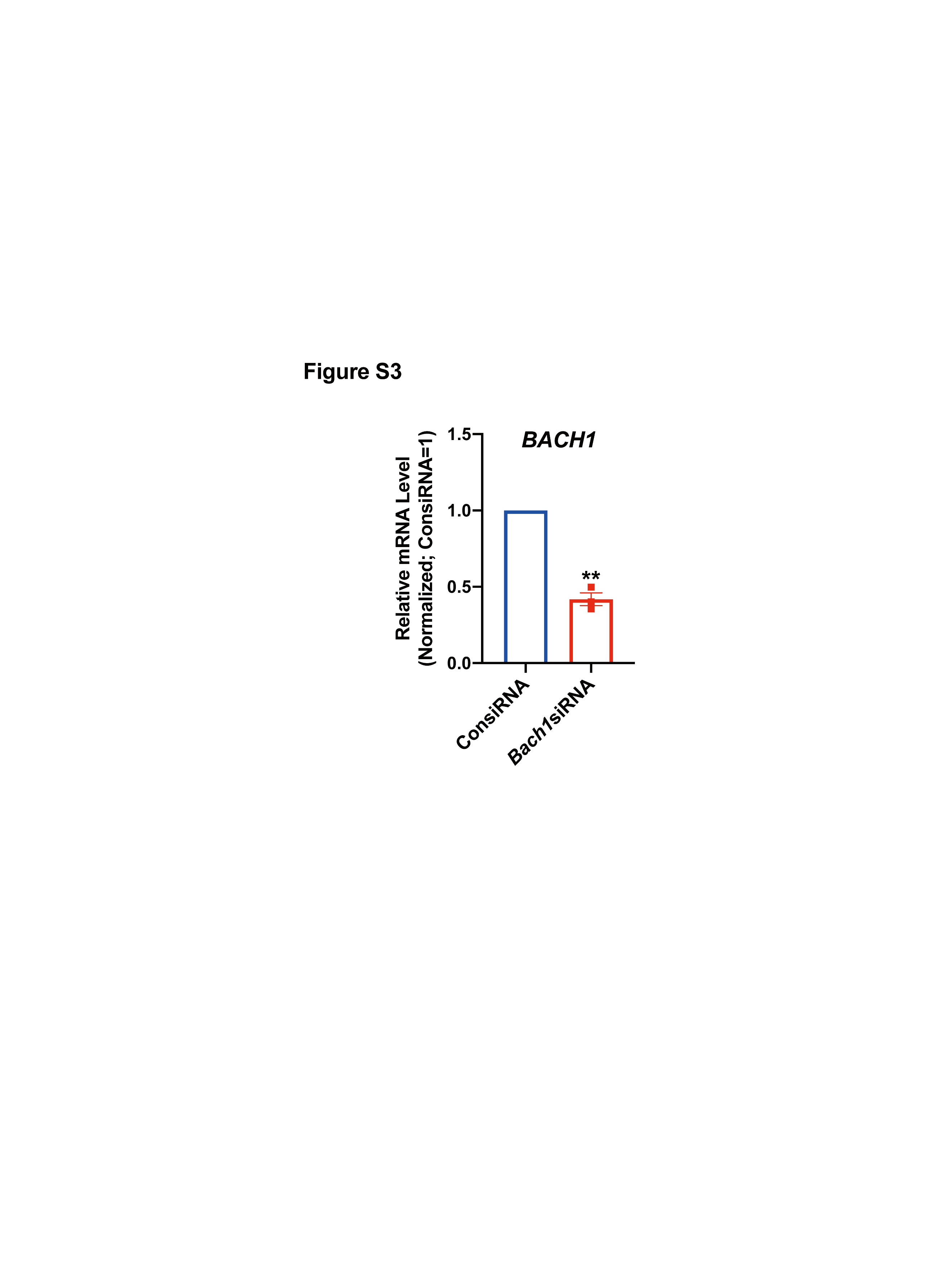
**

**Figure S3. qRT-PCR analysis of *BACH1* mRNA levels in HUVECs transfected with *Bach1*siRNA or ConsiRNA** (n=3 independent experiments, data are mean ± SEM, unpaired 2-tailed *t*-test, **p*<0.05, ***p*<0.01).
